# Management of small bowel obstruction and systematic review of treatment without nasogastric tube decompression

**DOI:** 10.1016/j.sopen.2022.10.002

**Published:** 2022-11-07

**Authors:** Kyle D. Klingbeil, James X. Wu, Antonia Osuna-Garcia, Edward H. Livingston

**Affiliations:** a Department of Surgery, UCLA School of Medicine, United States of America; b UCLA Biomedical Library (Louise M. Darling), United States of America

**Keywords:** Intestinal obstruction, Decompression, Surgical, Nasogastric tube, Small bowel obstruction, Water soluble contrast, Nasoenteric tube

## Abstract

**Background:**

Small bowel obstruction (SBO) is common and its management has evolved in recent years.

**Study design:**

The literature describing adhesive small bowel obstruction (aSBO) treatment was reviewed, and a formal systematic review was performed to identify publications reporting results of aSBO treatment without NGTs.

**Results:**

The annual rate of hospital admission for SBO in the US has increased, with 340,100 admissions in 2019 alone. SBO is usually treated with bowel rest, intravenous hydration and NGT placement. In recent years, water soluble contrast (WSC) has been used as a cathartic to simulate bowel function and may reduce hospital length of stay (HLOS) by 1.95 days (95%CI 0.56–3.3). There were 3 articles of the initial 1650 screened that reported outcomes of SBO treatment without NGTs. These articles included 759 patients, of whom 272 (36%) with aSBO were managed successfully without NGTs. When comparing outcomes to patients who did receive NGT decompression, there were no significant differences in operative rates (28.6% v 16.5%, risk ratio 1.34, 95% CI 1.0, 1.8). Mortality and rates of bowel resection were also not affected by NGT decompression (risk ratio 1.98, 95% CI 0.43, 9.10 and risk ratio 1.56, 95% CI 0.92, 2.65, respectively).

**Conclusion:**

SBO is a common disease process with increasing annual incidence. Use of WSC stimulates the bowel and may reduce HLOS. Modern aSBO treatment protocols should include NGT decompression with consideration of WSC administration. Selection of patients for treatment without NGT decompression requires further investigation.

## Introduction

Despite how frequent small bowel obstruction (SBO) is encountered, its optimal management remains elusive. Patients who have SBO may present with abdominal pain, nausea, emesis and abdominal distension. Patients with SBO are usually hospitalized in an acute care setting, given intravenous hydration, bowel rest and may or may not have a nasogastric tube (NGT) placed. If an NGT is placed, recent guidelines suggest that WSC should be administered to stimulate bowel activity. Although NGT placement is standard treatment for SBO, some patients refuse insertion because of its associated discomfort. Additionally, patients with altered anatomy may not be amenable to NGT placement. Is NGT decompression required for all patients with aSBO? Can patients with aSBO be treated safely without the use of NGT decompression? No current guidelines include the option to treat patients without NG decompression and the literature on this approach has not been systematically reviewed. The purpose of this article is to review the current management trends of aSBO with specific emphasis on its treatment without the use of NGTs. We hypothesize a subset of patients presenting with aSBO may be safely treated without the use of NGT decompression.

## Methods

Pubmed and Google Scholar were searched using the major headings of this review and the search terms, “small bowel obstruction”, “fluid replacement therapy”, “nasogastric tubes”, and “water soluble contrast”. References used for this review were also obtained from citations used in other publications related to bowel obstruction.

Regarding the systematic review examining treatment of aSBO without NGTs, search strategies were developed by a health sciences librarian (AOG) who translated the search concepts using each database platform's syntax, including search fields and field tags. The following databases was searched using the aforementioned strategies: PubMed (includes Medline), Embase, Web of Science Core Collection, and Cochrane Reviews and Trials.

For the search terms, MeSH, Emtree, and keywords were used for the concepts of “intestinal obstruction” and “nasogastric tube placement.” All concepts were combined with the “AND” Boolean operator. A full listing of the search strategy is presented in the supplement. A date limit was applied to each search strategy to obtain articles published from the databases inception to March 24, 2022. The search was limited to the English language. The references were downloaded for deduplication, screening, and appraisal.

Studies were included if they reported the effect of NGTs on aSBO for operative rates and/or hospital length of stay (HLOS). To be included, studies were required to have radiologic confirmation of aSBO from presumptive adhesive disease by plain films or CT. Studies were excluded if patients received long tube intubation (nasoduodenal, nasojejunal, e.g.). Only studies of presumptive adhesive disease were included by excluding studies reporting results in patients who had a diagnosis of idiopathic or postoperative ileus, an acute abdomen, malignant bowel obstruction, prior abdominal radiation, internal or external abdominal hernia or had prior abdominal surgery within six weeks. More detail about this search can be found in the supplement.

## Epidemiology

**Fig. 1:**
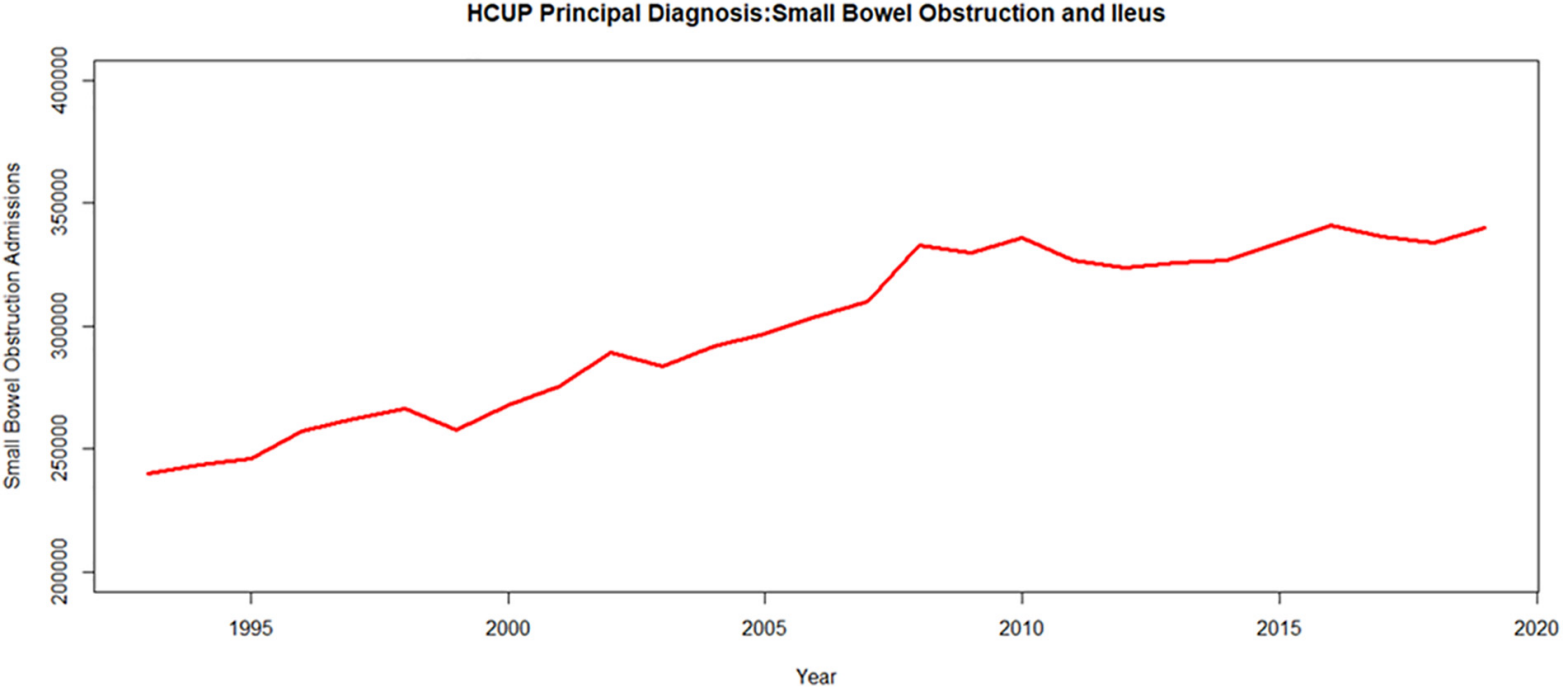
Annual incidence of US hospital admissions 1993–2019^⁎^. ^⁎^- Data derived from the Heath Care Utilization Project (HCUP). https://hcupnet.ahrq.gov/#setup and https://datatools.ahrq.gov/hcupnet. Temporal trends up to 2014 were based on a hospital admission being associated with a primary diagnostic ICD-9 code of 260.x (intestinal obstruction). After 2016, SBO was identified by the Clinical Classification (CCSR) DIG012-Intestinal Obstruction and Ileus. Data for 2015 were imputed because of missing data attributable to a change in coding systems from ICD-9 to ICD-10 that occurred in that year.

The annual incidence of US hospital admissions for SBO gradually increased until 2009, and has since reached a steady state (Fig. 1) [1]. In 2019, there were 340,100 US hospital admissions for SBO. The average length of stay for these patients was 5.0 days with an associated 1.5% inpatient mortality rate. SBO hospitalizations accounted for $4.1 billion in estimated costs to the healthcare system in 2019.

In one study of 150 patients admitted to the hospital with bowel obstruction, approximately ¾ of them were for SBO and ¼ for large bowel obstruction. Nearly 2/3 of bowel obstructions were caused by adhesions, 15% were caused by hernias and 13% by large bowel cancer. Surgery was required in 40% of patients [2].

## Pathophysiology

When the bowel is obstructed, intraluminal pressure increases, compromising intestinal venous outflow resulting in bowel wall edema. When intraluminal pressure exceeds systolic pressures, arterial blood flow becomes compromised, resulting in ischemia [3]. Colonic obstructions are especially prone to high intraluminal pressures in the presence of an intact ileocecal valve, resulting in a functional closed loop obstruction [4].

Two-thirds of accumulated gas in SBO is from swallowed air. The remainder is composed of CO2 diffused into the intestinal lumen from blood (2/3) and gas from bacterial fermentation (1/3). Removal of swallowed air is one of the main benefits of NGT decompression [4].

Peristalsis persists proximal to an obstruction for at least 7 days in animal experiments. The bowel wall becomes edematous and the bowel shortens in length by about 1/3. Distended bowel loses its mucosal integrity, contributing to fluid loss via the bowel lumen and diminished absorptive capacity [4,5].

## History of gastrointestinal drainage tubes

One of the most significant advances in the treatment of SBO occurred in the 1930's when Wangensteen demonstrated the benefits of gastrointestinal decompression [4,6]. Levin had developed a soft, single lumen tube in 1921 that could be passed into the duodenum through the nose [7]. The tubes original intent was to sample duodenal secretions in an attempt to diagnose gallbladder disease. The Levin nasoduodenal tube was adopted by Wangensteen who attached it to a suction device. These tubes were advanced along the small bowel to lie just above the point of obstruction [4,5,8]. Evacuation of air and fluid from the small intestine improved outcomes so greatly, that gastrointestinal intubation was universally adopted as the standard treatment for SBO. For the remainder of the 20th century, SBO treatment research was dominated by studies of different types of tubes, where they were placed within the bowel and how effectively they removed fluid from the intestinal lumen. A large body of literature accumulated advocating for placement of long tubes such as the Miller-Abbot long tube. These were inserted in the intestine, resting just above the obstruction itself [9,10].

Wangensteen and Levin are often credited for innovating the NGT. In fact, they studied nasoenteric and not nasogastric tubes [7,8]. In the years that followed Wangensteen's influential studies, it was believed that the key for successful non-operative treatment of SBO required suction of fluid away from the obstruction as close to the offending lesion as possible. Upper GI obstructions were treated with nasogastric tubes (NGTs) and distal obstructions with nasoenteric tubes. However, RCTs published in the 1990s showed no benefit of long tubes relative to NGTs [11]. Subsequently, NGTs have become the standard treatment for SBO and provide benefit, in part, from the removal of swallowed air.

## Clinical presentation

Patients who have SBO may present with a combination of abdominal pain, nausea, emesis and abdominal distension.

### Pain

Inflammation and obstruction of the appendix, small bowel, urinary and biliary tracts are all associated with crampy abdominal pain with minimal pain observed between cramps. Pain from SBO is felt in the mid abdomen. Establishing a history of crampy pain is important because constant, non-crampy pain may be associated with other etiologies, including pancreatitis and perforated viscus. Biliary colic radiates to the tip of the scapula and renal pain to the flank. Bowel ischemia may be present when pain is experienced between individual waves of cramps [4].

### Emesis

In his 1937 monograph about bowel obstruction, Wagensteen noted different patterns of emesis based on the location of a bowel obstruction. Colon obstruction is associated with crampy abdominal pain as a major feature with little vomiting. Placement of a nasogastric tube has little effect. In contrast, SBO is associated with frequent vomiting. After vomiting, a patient may feel better but intermittent crampy abdominal pain persists. Gastric obstruction is associated with little pain but frequent, non-bilious vomiting [4].

### Physical examination

Abdominal tenderness is common and, if severe, may be an indication for surgery. Patients with distended bowel will always have some element of tenderness, but that may not signal the presence of ischemic bowel. If the tenderness is severe and is associated with systemic toxicity, there is a high likelihood for bowel ischemia and laparotomy is warranted. Systemic toxicity is associated with fever, leukocytosis, and hypotension. Nausea is common and, if associated with vomiting, warrants placement of an NGT. Pain generally resolves along with successful bowel decompression.

## Diagnosis

A clinical presentation that includes recent onset of abdominal distension, nausea and vomiting is highly suggestive of SBO. Abdominal series will show dilated loops of small bowel. The presence of air in the rectum signifies a partial obstruction, whereas the absence of rectal air defines a complete obstruction. In the modern era, most diagnoses are made by CT imaging given the large amount of information available from a CT scan. CT imaging may demonstrate bowel dilation, in addition to alterations in bowel wall enhancement, bowel wall edema or hemorrhage, mesenteric edema, fat stranding, inter-loop abscesses, free fluid, the presence of multiple transition points (ie closed loop obstruction), or swirling of mesenteric vessels (ie “swirl” or “whirl” sign) suggestive of volvulus. Other findings, including bowel pneumatosis, mesenteric and/or portal venous gas, and free intraperitoneal air are more easily detected with CT imaging as well.

## Treatment

### Intravenous hydration

In the 1800's, the mortality from SBO was about 70% with or without surgery [5]. One of the first great advances in the treatment of SBO was the recognition that high SBO associated with substantial amounts of emesis was associated with dehydration and hypochloremia. Volume and electrolyte replacement with saline reduced but did not eliminate mortality from SBO.

When there is large volume gastric fluid loss from vomiting or NGTs, hypochloremic, hypokalemic metabolic alkalosis is frequently present. Carbonic anhydrase of the parietal cells secretes hydrochloric acid (140–160 meq/L) in large volume into the stomach. The bicarbonate made along with the acid is eventually secreted into the duodenal lumen, neutralizing the acid as it exits the stomach. When this cycle is interrupted by vomiting or nasogastric suction, balance is lost resulting in hypochloremic alkalosis. When overall fluid volume is preserved, the excess bicarbonate is secreted into the urine by the kidney. However, volume depletion usually occurs under these circumstances, activating the renin-angiotensin-aldosterone system that mediates increase renal bicarbonate reabsorption and potassium loss, potassium being lost in the urine in an effort to preserve hydrogen ions that are resorbed by the kidney [12,13].

Chloride replacement is essential when treating metabolic alkalosis caused by gastric fluid loss. Chloride deficit results in insufficient chloride to support bicarbonate exchange in the renal medullary collecting duct, causing bicarbonate resorption. When chloride is replaced, it becomes available to support bicarbonate exchange with resultant renal bicarbonate secretion and subsequent correction of the alkalosis [13]. This physiology explains why it is essential to replace gastric losses with chloride containing normal saline. Potassium is lost by the kidney secondary to an aldosterone-mediated exchange between potassium and hydrogen ion. Hypokalemia should be corrected by careful administration of oral or intravenous potassium boluses [12]. Care should be taken when choosing the type of resuscitative fluid. Balanced salt solutions that have lactate, acetate or gluconate can worsen alkalosis in patients with renal dysfunction or who are sodium depleted [12].

When SBO results in large volume loss of alkali-rich small bowel contents, volume contraction and acidosis occur. In this situation, bicarbonate should be replaced Ringer's lactate, an isotonic (273 mOsm/L) solution having a pH = 6.5 composed of sodium (130 mEq), chloride (109 mEq), lactate (28 mEq), potassium (4 mEq) amd calcium (2 mEq). Hepatic metabolism of lactate to bicarbonate replaces lost bicarbonate. When there are large, ongoing fluid losses from the GI tract, it is a good practice to measure the electrolyte concentration of the fluid and then adjust the electrolyte composition of intravenous resuscitation fluids accordingly.

### Nasogastric tubes

In the absence of bowel compromise, bowel rest, fluid repletion and placement of an NGT are the initial treatments for SBO. In recent years, intestinal stimulation with WSC through an NGT has gained increasing acceptance as a treatment for aSBO [14,15]. A recent systematic review and meta-analysis of the effect of WSC for the treatment of aSBO revealed that use of WSC via an NGT shortly after a patients was admitted to the hospital with a diagnosis of aSBO decreased the hospital length of stay by 1.95 days (95%CI 0.56–3.3) [16]. The use of WSC did not affect the risks of bowel resection, complications, or mortality. Additional outcomes could not be assessed given insufficient reporting of associated outcomes. Although promising, the body of literature supporting the use of WSC for the treatment of aSBO remains limited because of substantial heterogeneity and low quality of the studies published to date. However, if WSC is as effective as these studies suggest and it can reduce hospital length of stay by 2 days, there would be a potential savings of about $2 billion to the health care system [1].

WSC stimulates bowel activity because of its hyperosmolar properties. Gastrografin® (GG) is the most widely used and studied WSC agent. It is a mixture of nonabsorbable sodium diatrizoate and meglumine diatrizoate that has an osmolarity of 1900 mOsm/L [17]. The 6-fold higher osmolarity relative to serum (normally osmolarity ranges from 275 to 295 mOsm/L) provides an osmotic pressure gradient to drive water from within the bowel wall to the intraluminal space. This, in turn, reduces bowel wall edema, restores normal blood flow and facilitates smooth muscle contractility [18]. The dilution of bowel contents is also thought to promote its passage past the site of obstruction [19]. The combination of these physiological effects provides therapeutic benefit by shortening the duration of aSBO and preventing the need for surgery. Other derivative WSC agents, including Urografin® [20] and MD-Gastroview® [21], have also been used for the treatment of aSBO.

The use of barium (lipid soluble) contrast is associated with the theoretical risk of peritonitis in the setting of bowel perforation, although the level of evidence supporting this risk is low [22]. WSC is therefore the preferred oral contrast agent for radiographic gastrointestinal studies given that if extravasated, it will not cause peritonitis. The opposite is true when there is concern for aspiration. Barium aspiration is well tolerated and often patients are asymptomatic [[23], [24], [25]]. The high osmolarity of WSC creates a pressure gradient leading to rapid fluid shifts into the tracheobronchial lumen resulting in pulmonary edema and respiratory failure [26]. Previous studies have examined the benefit of barium contrast for the treatment of aSBO, however these studies either did not stratify outcomes based on barium vs. WSC [27] or used a mixture of barium and WSC [28], yielding negative results. Previous studies of WSC for the treatment of aSBO reported few cases of pulmonary complications, suggesting that in the setting of a functioning NGT, WSC administration is safe [16]. Administration of WSC in the setting of an aSBO without an NGT therefore cannot be recommended given the risks of aspiration-related complications.

### Treatment of aSBO without NGT decompression

NGTs are associated with substantial pain and discomfort [[29], [30], [31], [32], [33]]. This has led some clinicians and patients to avoid their use. Additionally, to function properly, NGTs require expert management. It is relatively common when seeing hospitalized patients to find that their NGTs are not functioning correctly. When NGTs become occluded, clamped, or assembled improperly, they stent open the esophagus and can increase the risk of aspiration. To investigate experience with managing aSBO without NGTs, we performed a systematic review (Supplement). There were 3 retrospective studies reporting on hospital experiences in 759 patients treating aSBO that included patients treated without NGTs [[34], [35], [36]]. In these series, 36% (n = 292) of patients were managed without an NGT. No major complications were associated with treating aSBO without an NGT. There was no effect of NGT placement on operative, bowel resection or mortality rates. The risk of aspiration is believed to be high in patients presenting with SBO, and that NGT decompression reduces this risk. However, in the series we reviewed, there was no increased risks of pulmonary complications when aSBO was treated without NGT decompression. Although it may be possible to treat aSBO without NGTs in some patients, it is not known how to identify such patients and further research is needed to better understand this approach. The complete results of the systematic review and meta-analysis are found in the supplement.

## Guidelines

Guideline recommended treatment for aSBO includes bowel rest, IV hydration and placement of an NGT [14,15]. Patients are observed until the subjective sensation of flatus signals that bowel function has been restored. If the aSBO does not resolve, laparotomy is required. Although it is believed that prompt surgery results in better outcomes, there is little agreement on when to declare failure of non-operative observation. Similarly, if observation is successful and a diet is resumed, how to optimally resume a diet is not supported by evidence. Cathartics such as WSC facilitate resumption of bowel function and their use can be considered.

There are at least 2 guidelines for the management of SBO that recommend WSC studies. The 2017 Bolgna guideline was written by the Adhesive Small Bowel Obstruction group of the World Society of Emergency Surgery [14]. They report performing a systematic search of the literature but present only 3 references to support their recommendation. These were systematic reviews dating from 2005 to 2016 [[37], [38], [39]]. No quality assessment of the reviewed literature was provided and the recommendation for the use of WSC was vague. The guideline states, “If the contrast has not reached the colon on an abdominal X-ray taken 24 hours following administration of the contrast, this is highly indicative of failure of non-operative management.” No specification was provided for when the WSC study should be done relative to hospital admission. The guideline mentioned, but did not explore, WSC's ability to stimulate the bowel, contributing to a therapeutic effect in treating aSBO.

The Eastern Association for the Surgery of Trauma published guidelines in 2012 regarding the management of SBO. A PubMed search from 2007 to 2011 was performed and reviewed by at least 2 of 10 acute care surgeons who developed the guideline [15]. They recommended “Water-soluble contrast should be considered in the setting of partial aSBO that has not resolved in 48 hours because it can improve bowel function (time to bowel movement), decrease HLOS, and is both therapeutic and diagnostic. Level 2.” Level 2 was defined as “This recommendation is reasonably justifiable by available scientific evidence and strongly supported by expert critical care opinion.”

Neither guideline fulfilled all the Institute of Medicine criteria for guideline quality [40]. Additionally, both were based on an incomplete assessment of the literature and lacked critical analysis of the quality of studies they relied on.

## Limitations

This review has several limitations, 1) the literature basis consists mostly of retrospective cohort studies that may be affected by selection bias, eg, those with worrisome radiographic imaging, vital instability or significant abdominal pain may have an NGT placed more frequently to reduce enteric luminal volume to improve the manipulation of organs if surgical intervention is required; 2) detailed treatment protocols were lacking in the studies reviewed; 3) complication rates were incompletely reported; 4) baseline patient characteristics were not balanced; 5) patient cohorts were not necessarily generalizable given that most of the studies were from single centers; 6) no study provided quality of life as a study outcome.

Conclusion?

## Data access

All data collected for this research are reported in the manuscript and accompanying supplement.

## Ethics approval

As a review of published literature that did not involve individual patients, this work did not require IRB approval.

## Funding sources

UCLA Department of Surgery Research Funds.

## CRediT authorship contribution statement

Kyle D Klingbeil MD MS- Data curation; Investigation; Validation; Writing - review & editing.

James X Wu MD- Investigation; Validation; Writing - review & editing.

Antonia Osuna-Garcia, MLIS- Data curation; Writing - review & editing.

Edward H Livingston MD- Conceptualization; Data curation; Formal analysis; Funding acquisition; Investigation; Methodology; Project administration; Resources; Software; Supervision; Validation; Visualization; Roles/Writing - original draft; Writing - review & editing.

## Declaration of competing interest

None of the authors have any relevant financial conflict of interest relevant to the submitted publication.

## Appendix A Supplementary data

Supplementary material Image 1
