# Supplementary material for: Management of small bowel obstruction and systematic review of treatment without nasogastric tube decompression
Source: Surg Open Sci. 2022 Nov 7;12:62–7. doi: 10.1016/j.sopen.2022.10.002 (PMC10040372; doi:10.1016/j.sopen.2022.10.002)
Supplement: Supplementary file 1 — Supplementary material [file mmc1.docx]

**SUPPLEMENT**

**Systematic Review: Treating SBO without NGTs**

**METHODS**

**Study Screening and Selection Criteria (inclusion/exclusion criteria)**

The search strategies were developed by a health sciences librarian (AOG) who translated the search concepts using each database platform’s syntax, including search fields and field tags. The following databases was searched using the aforementioned strategies: PubMed (includes Medline), Embase, Web of Science Core Collection, and Cochrane Reviews and Trials.

For the search terms, MeSH, Emtree, and keywords were used for the concepts of “intestinal obstruction” and “Nasogastric tube placement.” All concepts were combined with the “AND” Boolean operator. A full listing of the search strategy is presented in the supplement. A date limit was applied to each search strategy to obtain articles published from the databases inception to March 24, 2022. The search was limited to the English language. The references were downloaded for deduplication, screening, and appraisal.

Studies were included if they reported the effect of NGTs on SBO for operative rates and/or hospital length of stay (HLOS). To be included, studies were required to have radiologic confirmation of SBO from presumptive adhesive disease by plain films or CT. Studies were excluded if patients received long tube intubation (nasoduodenal, nasojejunal, e.g.). Only studies of presumptive adhesive disease were included by excluding studies reporting results in patients who had a diagnosis of idiopathic or postoperative ileus, an acute abdomen, malignant bowel obstruction, prior abdominal radiation, internal or external abdominal hernia or had prior abdominal surgery within six weeks.

**Outcome Measures, Interventions, and Comparison Groups**

We assessed 1) The proportion of patients not getting an NGT upon diagnosis of SBO, 2) outcomes for NGT versus non-NGT, 3) treatment failures defined by the number of patients initially treated without an NGT who eventually have an NGT placed, 4) the incidence of surgery, 5) the incidence of pneumonia, 6) the incidence of persistent vomiting, 7) hospital length of Stay, 8) quality of life, 9) use of decision-making protocols for NG placement, when to perform surgery and discharge patients from the hospital.

**Data Extraction**

Two investigators (EHL and KDK) independently reviewed the studies the medical librarian identified for potential inclusion. The following information was extracted from each study (if appropriate): trial design characteristics (randomization procedure and blinding), descriptions of the experimental and control groups, baseline characteristics of the patients, eligibility criteria for NGT placement, and trial location. If available, sample size calculations and the rationale for selection of minimal clinically important differences were reviewed. For each study, two reviewers (EHL and KDK) independently assessed the risk of bias for the outcomes of interest. Disagreements were resolved by consensus or by additional review by a third investigator (JXW).

**Appraisal of Individual Studies**

The quality of the reviewed literature was assessed by the Newcastle-Ottawa scale for observational research.^24^ Observational studies were also evaluated using a modified STROBE criteria (supplement) when assessing studies of NGT treatment of SBO.

A spreadsheet was created containing data fields necessary for the Newcastle-Ottawa quality tool. One investigator filled out this spreadsheet (KDK). The accuracy of the data extraction was verified by EHL. An excel file was used to catalog data used for the Cochrane Risk of Bias 2 instrument (<https://www.riskofbias.info/welcome/rob-2-0-tool/current-version-of-rob-2>).

**Evaluation of the Evidence. Quantitative and Qualitative Synthesis Strategies**

For outcomes with available data (HLOS and operative rates), a random effects meta-analysis was performed to summarize treatment effects using absolute risk differences.^25^ A random effects model was used because the effect of various treatments for SBO may differ from one center from another because of local differences in treatment protocols and unmeasurable patient characteristics. Treatment effects across the observational studies were estimated and summarized on an absolute risk difference scale. Heterogeneity across the observational studies was assessed by using I^2^ and τ metrics^26^. HLOS, pneumonia rates and the proportion of patients undergoing surgery were displayed as forest plots. Where heterogeneity precludes meta-analysis, a qualitative synthesis of the studies was performed.

**RESULTS**

**STUDY CHARACTERISTICS**

There were 1,650 articles obtained in the initial search. There were 390 duplicates, leaving 1,260 references for title/abstract review. Of these remaining 1,232 were excluded because they were believed to not be relevant based on review of the title and abstract or had keywords suggesting that these articles were not relevant to the current review (Supplement). Full text review was performed on the remaining 28 articles. After manual review of the full text articles, 25 were found to not fulfill inclusion criteria for this review, leaving three articles for the final analysis. The included studies were published between 2013 and 2021 and included a total of 759 patients. Of these, 272 (36%) patients had their SBO managed without NGTs. Two studies were located in the US^27,28^, and the remaining study from Japan^29^. The included studies were retrospective cohort analyses. No randomized trials were found (Table 1).

**STUDY QUALITY**

**Newcastle-Ottawa Quality Assessment Tool for Observational Research and Additional Quality Measures**

The Newcastle-Ottawa quality assessment scored all observational studies as having a ‘high risk of bias’ (Table 2). Studies were downgraded for multiple limitations. All included studies lacked generalizability as single center studies, and the majority failed to describe how diagnosis and treatment with NGT was ascertained. Studies did not attempt to balance patient baseline characteristics between groups. There was also a lack of independent blind assessment of outcomes. All included studies did, however, provide adequate follow-up and used medical record linkage for the assessment of outcomes.

The enhanced STROBE instrument was also used to assess quality within the included studies (Table 3). Two of the three studies declared a study hypothesis. One of these asked a discrete research question (if NGT would decrease rate of emesis)^29^. Included studies failed to completely outline eligibility and exclusion criteria and lacked thorough descriptions of included outcomes, confounders, and effect modifiers. None of the included studies addressed potential bias nor did they describe sample size calculations. Statistical methods were not well described and no explanations were provided as to how missing data was addressed. Two of the three studies provided a conflict-of-interest (COI) statement and source of funding, with no studies demonstrating potential major conflicts of interest.

**Guidelines**

There are no guidelines available in the current literature to describe patient populations who may be treated without NGT decompression. The Bologna guidelines recommend NGT decompression as part of the standard treatment for adhesive SBO^30^.

**INTERVENTION/EXPOSURE**

The diagnosis of SBO was made using a combination of clinical examination and imaging assessment. CT imaging was used in all studies. NGT placement occurred immediately after the diagnosis of SBO was made in most patients. One study indicated a sub-group of patients who had an NGT placed 6 hours after diagnosis (n=9) compared to those who received an NGT immediately (n=162)^27^.

No studies described protocols determining when surgery would be performed, when NGTs were removed / diet resumed and when patients could be discharged from the hospital. Two studies indicated a clearly defined primary outcome variable (POV).

**OUTCOMES**

**Effect of NGT on HLOS**

All three studies included HLOS for all patients, including those who underwent surgery, as an outcome variable. One study included a sub-group analysis of HLOS excluding patients who underwent surgery^27^.

Only one study reported dispersion data such as standard deviations or interquartile ranges for HLOS, precluding the ability to perform a meta-analysis. Time from resolution of SBO to hospital discharge was not reported.

Three observational studies reported HLOS for patients who did not undergo surgery, however dispersion data was not provided.

**Effect of NGT on Operative Rates**

Operative rates were reported in all included studies. In total, of the 468 patients who received an NGT, 134 (28.6%) underwent surgery. Of the 291 patients who did not receive an NGT, 48 (16.5%) underwent surgery. Random effects meta-analysis showed no association between treatment with NGT or control (no NGT) for the patients who underwent surgery (risk ratio 1.34, 95% CI 1.0, 1.8) (Supplement Figure 1).

Operative findings were described in two studies, including rates of bowel resection and bowel necrosis ^27,28^. Random effects meta-analysis showed no association between treatment with NGT or control (no NGT) on bowel resection (risk ratio 1.56, 95% CI 0.92, 2.65) (Supplement Figure 2). Negative laparotomy rates were not reported.

**Other outcomes**

Time to resolution, defined by first meal, was reported in two studies^27,29^. The definitions for initial time points were not provided. Treatment failure rate (defined as placement of NGT in non-NGT group) was only described in one study^29^. Water soluble contrast (WSC) studies were not included or reported in the included studies.

Overall complication rates were provided in one study^27^ and mortality rates were reported in two studies^28,29^. Random effects meta-analysis showed no association between treatment with NGT or control (no NGT) on mortality (risk ratio 1.98, 95% CI 0.43, 9.10) (Supplement Figure 3). The rate of NGT-specific complications, including pneumonia and respiratory failure, were described two studies^27,29^. Emesis rates between groups after initial treatment was described in one study^29^. Patient satisfaction was not measured in any of the included studies.

Meta-analysis of time to resolution, overall complications and pneumonia rates could not be performed because there was insufficient information provided about these outcomes in the reviewed articles.

Table 1. Summary of the reviewed studies.

| Author | Year | Study Design | Center Type (No.) | No. Patients without NGT | No. Patients with NGT | HLOS  Patients without NGT | HLOS  Patients with NGT | Time to resolution Patients without NGT | Time to resolution Patients without NGT | Operative Rate Patients without NGT (%) | Operative Rate Patients with NGT (%) | Mortality Patients without NGT (%) | Mortality Patients without NGT (%) | Pneumonia rate Patients with NGT (%) | Pneumonia rate Patients without NGT (%) | Emesis rate Patients with NGT (%) | Emesis rate Patients without NGT (%) |
| --- | --- | --- | --- | --- | --- | --- | --- | --- | --- | --- | --- | --- | --- | --- | --- | --- | --- |
| Fonseca | 2013 | Retro spective Cohort | Single | 55 | 235 | 3.18 (-) | 10.16 | 1.63 | 2.97 | 17 (30.9) | 87 (37.0) | - | - | 0 | 16 (11.6) | - | - |
| Berman | 2017 | Retro spective Cohort | Single | 88 | 93 | 4.2 (-) | 7 | - | - | 20 (22.7) | 29 (31.2) | 2 (2.3) | 3 (3.2) | - | - | - | - |
| Shinohara | 2021 | Retro spective Cohort | Single | 148 | 140 | 8 (1-55)^ | 10 (2-99)^ | 4 (1-21)^ | 5 (2-36)^ | 11 (7.43) | 18 (12.9) | 2 (1.4) | 0 | 2 (1.4) | 0 | 18 (12.9) | 28 (18.9) |

Table 2: Summary table of included studies comparing NGT to no-NGT in the treatment for SBO.

Numerical data displayed as mean (SD) unless otherwise specified.

^Median values (range)

- Indicates data not available

* Data only given as Odds Ratio (OR) and (95% confidence interval)

Table 2: New-Castle Ottawa Analysis for Included Cohort Studies


Table 3: STROBE Analysis for Included Cohort Studies

Search Strategy

Treating Small Bowel Obstruction Without Nasogastric Tubes

PubMed

Concept Controlled Vocab Keywords

Small bowel obstruction

Intestinal obstruction "Intestinal Obstruction"[Mesh:NoExp] OR "Intestinal Obstruction/diagnostic imaging"[Mesh:NoExp] OR "Intestinal Obstruction/drug therapy"[Mesh:NoExp] OR "Intestinal Obstruction/surgery"[Mesh:NoExp]

OR

"Intestine, Small"[Mesh:NoExp] OR "Intestine, Small/diagnostic imaging"[Mesh:NoExp] OR "Intestine, Small/surgery"[Mesh:NoExp]

OR

"Tissue Adhesions"[Mesh:NoExp] OR "Tissue Adhesions/diagnostic imaging"[Mesh:NoExp] OR "Tissue Adhesions/drug therapy"[Mesh:NoExp] OR "Tissue Adhesions/surgery"[Mesh:NoExp] ((Intestin*[Title/Abstract] OR bowel[Title/Abstract] OR “small bowel”[Title/Abstract] OR “small intestine”[Title/Abstract] OR tissue[Title/Abstract])

AND

(Obstruct*[Title/Abstract] OR adhesion*[Title/Abstract]))

OR SBO[Title/Abstract]

Nasogastric tube decompression

"Decompression, Surgical"[Mesh:NoExp] OR "Decompression, Surgical/methods"[Mesh:NoExp] OR "Decompression, Surgical/statistics and numerical data"[Mesh:NoExp]

((Nasogastric[Title/Abstract] OR “nasogastric tube”[Title/Abstract]) AND (decompression[Title/Abstract] OR placement[Title/Abstract] OR intubation[Title/Abstract]))

Results Limits: none

Results: 513

All relevant articles included

Searched on 3/22/2022 Relevant articles:

34872712[PMID]

23574854[PMID]

28912083[PMID]

Final Pubmed search string:

("Intestinal Obstruction"[MeSH Terms:noexp] OR "intestinal obstruction/diagnostic imaging"[MeSH Terms:noexp] OR "intestinal obstruction/drug therapy"[MeSH Terms:noexp] OR "intestinal obstruction/surgery"[MeSH Terms:noexp] OR "intestine, small"[MeSH Terms:noexp] OR "intestine, small/diagnostic imaging"[MeSH Terms:noexp] OR "intestine, small/surgery"[MeSH Terms:noexp] OR "Tissue Adhesions"[MeSH Terms:noexp] OR "tissue adhesions/diagnostic imaging"[MeSH Terms:noexp] OR "tissue adhesions/drug therapy"[MeSH Terms:noexp] OR "tissue adhesions/surgery"[MeSH Terms:noexp] OR (("intestin*"[Title/Abstract] OR "bowel"[Title/Abstract] OR "small bowel"[Title/Abstract] OR "small intestine"[Title/Abstract] OR "tissue"[Title/Abstract]) AND ("obstruct*"[Title/Abstract] OR "adhesion*"[Title/Abstract])) OR "SBO"[Title/Abstract]) AND ("decompression, surgical"[MeSH Terms:noexp] OR "decompression, surgical/methods"[MeSH Terms:noexp] OR "decompression, surgical/statistics and numerical data"[MeSH Terms:noexp] OR (("Nasogastric"[Title/Abstract] OR "nasogastric tube"[Title/Abstract]) AND ("decompression"[Title/Abstract] OR "placement"[Title/Abstract] OR "intubation"[Title/Abstract])))

Embase

Concept Controlled Vocabulary Keywords Notes

Intestinal obstruction 'intestine obstruction'/de OR

'small intestine'/de

OR

'tissue adhesion'/de ((Intestin*:ti,ab OR bowel:ti,ab OR “small bowel”:ti,ab OR “small intestine”:ti,ab OR tissue:ti,ab)

AND

(Obstruct*:ti,ab OR adhesion*:ti,ab))

OR SBO:ti,ab

Nasogastric tube decompression

"Decompression, Surgical"[Mesh:NoExp] OR "Decompression, Surgical/methods"[Mesh:NoExp] OR "Decompression, Surgical/statistics and numerical data"[Mesh:NoExp]

((Nasogastric:ti,ab OR ‘nasogastric tube’:ti,ab) AND (decompression:ti,ab OR placement:ti,ab OR intubation:ti,ab))

Results No limits or filters applied

Results: 945

Searched on: 3/22/2022

Final Embase search string:

('intestine obstruction'/de OR 'small intestine'/de OR 'tissue adhesion'/de OR ((intestin*:ti,ab OR bowel:ti,ab OR 'small bowel':ti,ab OR 'small intestine':ti,ab OR tissue:ti,ab) AND (obstruct*:ti,ab OR adhesion*:ti,ab)) OR sbo:ti,ab) AND ('decompression surgery'/de OR ((nasogastric:ti,ab OR 'nasogastric tube':ti,ab) AND (decompression:ti,ab OR placement:ti,ab OR intubation:ti,ab)))

Web of Science

Concept Keywords Notes

Intestinal obstruction ((Intestin*OR bowel OR “small bowel” OR “small intestine” OR tissue)

AND

(Obstruct* OR adhesion*))

OR SBO

Nasogastric decompression ((Nasogastric OR “nasogastric tube”) AND (decompression OR placement OR intubation)) OR “surgical decompression”

Results No limits or filters applied

Results: 123

Searched on: 3/22/2022

Final WOS search string:

((Intestine*OR bowel OR “small bowel” OR “small intestine” OR tissue) AND (Obstruct* OR adhesion*)) OR SBO (Topic) and ((Nasogastric OR “nasogastric tube”) AND (decompression OR placement OR intubation)) OR “surgical decompression” (Topic)

Cochrane Reviews and Trials

Searched on 3/22/2022

Cochrane Reviews Results: 2

Cochrane Trials Results: 67

No limits or filters applied.

ID Search Hits

#1 MeSH descriptor: [Intestinal Obstruction] this term only 361

#2 MeSH descriptor: [Intestinal Obstruction] this term only and with qualifier(s): [diagnostic imaging - DG, drug therapy - DT, surgery - SU] 164

#3 MeSH descriptor: [Intestine, Small] this term only 573

#4 MeSH descriptor: [Intestine, Small] this term only and with qualifier(s): [diagnostic imaging - DG, surgery - SU] 106

#5 MeSH descriptor: [Tissue Adhesions] this term only 532

#6 MeSH descriptor: [Tissue Adhesions] this term only and with qualifier(s): [diagnostic imaging - DG, drug therapy - DT, surgery - SU] 107

#7 (((Intestin* OR bowel OR “small bowel” OR “small intestine” OR tissue) AND (Obstruct* OR adhesion*)) OR SBO):ti,ab,kw 6350

#8 #1 OR #2 OR #3 OR #4 OR #5 OR #6 OR #7 6857

#9 MeSH descriptor: [Decompression, Surgical] this term only 531

#10 MeSH descriptor: [Decompression, Surgical] this term only and with qualifier(s): [methods - MT] 250

#11 MeSH descriptor: [Decompression, Surgical] this term only and with qualifier(s): [statistics & numerical data - SN] 24

#12 (((Nasogastric OR “nasogastric tube”) AND (decompression OR placement OR intubation))):ti,ab,kw 765

#13 #9 OR #10 OR #11 OR #12 1284

#14 #8 AND #13 69

Search de-duplication and filter by keywords

1650 references

After De-Duplication=1260

Exclude:

KW: Animal Studies

KW: Tumors

KW: Pancreatic Diseases

KW: Appendicitis

Title: Cocoon =1250

KW: Adenocarcinoma

KW: Midgut volvulus=

KW: Infant

KW: Endometriosis

KW: magnetic=1099

KW: intussusception

KW: Diverticulitis

KW: Rare Diseases

KW: *congenital

KW: Colonic Diseases=1046

KW: Tomography

KW: *Colonic Neoplasms

KW: Cell Adhesion

KW: Fecal Impaction

KW: Afferent Loop Syndrome=808

KW: Neoplasms

Year < 1980

KW: Child

KW: Volvulus

Title: Midgut volvulus=657

Notes: Letter

KW: Palliative Care

KW: Urinary Bladder

KW: *Colonoscopy= 652

KW: Pregnancy

KW: Cell Survival

Title: Hernia

Title: Constipation= 609

To hand search=

After hand title search=28

After full text review=3

Supplement Figure 1: Meta-analysis for Operative Rates


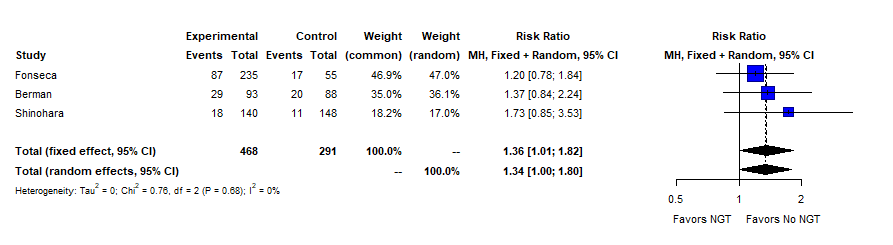


Supplement Figure 2: Meta-analysis for Bowel Resection


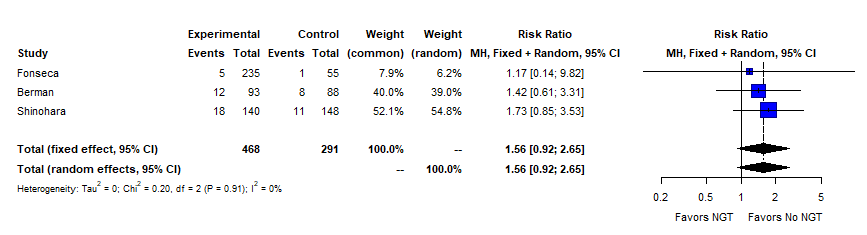


Supplement Figure 3: Meta-analysis for Mortality

**
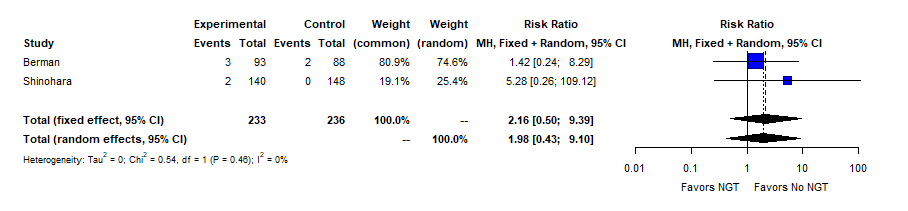
**
